# Prevalence and Pattern of Idiopathic Osteosclerosis and Condensing Osteitis in a Saudi Subpopulation

**DOI:** 10.7759/cureus.22234

**Published:** 2022-02-15

**Authors:** Mey A Al-Habib

**Affiliations:** 1 Department of Endodontics, Faculty of Dentistry, King Abdulaziz University, Jeddah, SAU

**Keywords:** prevalence, mandible, bony islands, condensing osteitis, idiopathic osteosclerosis

## Abstract

Background

This study aims to investigate the prevalence and pattern of idiopathic osteosclerosis (IO) and condensing osteitis (CO) in the jaws of a Saudi Arabian subpopulation with regards to gender, age, shape, localization, and tooth relationship.

Methodology

Digital panoramic and periapical radiographs with clinical data of 1,000 patients at King Abdulaziz University Dental Hospital were analyzed to determine the incidence of IO and CO lesions in relation to age, gender, location in the jaws, and dental status of the associated tooth. Descriptive statistics and chi-square tests were used for data analysis.

Results

Out of the 752 patients (495 females, 257 males), IO was identified in 66 (8.8%) patients, while 44 (5.9%) patients had CO. IO occurred more in females (68.2%) than males (31.8%). CO had a statistically significant higher rate of occurrence in females (81.8%) than males (18.2%) (P ≤ 0.05). IO and CO lesions were observed to be higher in individuals in their third decade, and 100% were in the mandibular molar area. Almost half of the identified IO (48.5%) had a rounded shape radiopacity, while CO mostly appeared irregular (63.6%).

Conclusions

The prevalence of IO and CO in the studied Saudi subpopulation was low. Both lesions were more frequent in females in their third decade and were primarily found in the mandibular molar region.

## Introduction

Idiopathic osteosclerosis (IO) is a benign sclerotic condition that can affect different areas of the body, including the mandible. The lesion is believed to be of unknown origin, hence the term “idiopathic,” and is also referred to as a dense bone island, bone eburnation, or bone enostosis [1-5]. IO develops as an asymptomatic, localized, homogenously radiopaque foci of dense bone, typically with distinct outlines that do not result from an infection or systemic disease [2]. Radiographically, the condition can take many different forms with variable size, shape, outline, and density [6]. It can be associated with the roots of the teeth or can be completely separated by the normal trabecular part of the alveolar bone. When the condition occurs in the periapical areas, it can expand far beyond the apices of the related teeth and can be found in the edentulous areas. On radiographs, when IO is near the apex of a tooth, the lesion presents as well-defined, densely uniform, and non-expanding radiopacity with normal periodontal space and lamina dura [7]. IO is classified into distinct categories according to its size and location. “Interradicular radiopacity” is usually confined to the area between the roots and is continuous with the lamina dura of at least one adjacent tooth. “Interradicular and separate radiopacity” is limited to the area between the roots and is separate from the lamina dura. “Apical and interradicular radiopacity” is usually recognized at the tooth apex with an extension between the roots, whereas an “apical radiopacity” is predominantly located at the root apex. A “separate radiopacity” is defined as an apical and distinctly isolated radiopacity from the teeth and lamina dura [4]. Some studies have demonstrated a relationship between traumatic occlusion and the development of IO [8]. It is generally believed that IO does not change in size over time on radiographs. However, multiple studies have shown size changes in dense bone islands, which have exhibited metabolic activity [4]. IO lesions can hinder both tooth eruption and orthodontic tooth movement. In addition, it can recur after surgical excision and persist for several years without remodeling [9].

Condensing osteitis (CO) is an asymptomatic, localized, radio-opaque jaw lesion that is usually discovered through routine radiographic examination. CO, also known as periapical sclerosing osteitis or focal sclerosing osteitis, occurs as a result of chronic pulpal infections of the teeth with deep caries or large restorations. Contrary to IO, the pulp in CO is affected, either inflamed or sometimes necrotic, for a very long time [10]. Solitary thickening of the trabeculae is usually observed within the marrow spaces adjacent to the roots of the affected teeth with sclerotic changes of the apical bone. This is an outcome of the adjacent bone trying to stop the inflammation or reform the bone impacted by the inflammatory process [11]. As a complication of CO, changes in the tooth alignment or challenges in orthodontic treatment can occur. An association between CO and root resorption has been described in the literature [12]. CO can occur anywhere in the jaw with a preference for the mandibular arch in the premolar/molar area [10].

CO mostly occurs as a bone response to a prolonged mild irritation that arises from a chronic inflammatory process caused by trauma from occlusion or pulpal disease; hence, the term CO. Alternatively, IO is usually considered developmental rather than reactive and presents as a normal anatomic bone variation formed during early bone development [13].

Other factors that can differentiate between these two conditions are either the location of the lesions, whether in the maxilla or the mandible, or the tooth relation, whether the lesion is related to the apex or interradicular space or separate from the associated tooth. In addition, they can be differentiated based on the shape and border of the lesion, whether it is round, oval, or irregular with well or ill-defined borders.

In approximately 70% of CO cases, performing endodontic treatment in pulpally involved teeth reverts bone density to normal. However, in some cases, the changes to the alveolar bone remain visible on radiographs, sometimes even after the associated tooth has been extracted [7].

Some of the differential diagnoses for both CO and IO include cementoblastoma, hypercementosis, root remnants of deciduous teeth, cementing fibroma, ossifying fibroma, or diffuse sclerosing osteomyelitis. Moreover, radiopacities in edentulous regions after tooth extraction may represent residual CO, or they can be excessively ossified regions formed after surgery [14].

To our knowledge, no prior studies have investigated the prevalence and characteristics of IO and CO simultaneously in a Saudi Arabian population. Hence, the primary goal of our study is to assess the overall prevalence and pattern of IO and CO in the jaws of a Saudi Arabian subpopulation with respect to age, gender, shape, localization, and dental relationship. In addition, this study will highlight common significant dental findings associated with CO and IO, such as deep caries, restoration, and root canal therapy, and aid in the differential diagnosis of both lesions.

## Materials and methods

This retrospective study was performed after obtaining ethical approval from the Research Ethics Committee at the Faculty of Dentistry, King Abdulaziz University (186-11-19). The study was conducted using available digital panoramic and periapical radiographs and the clinical information of 1,000 randomly selected patients attending routine appointments at the Faculty of Dentistry between 2013 and 2019. One board-certified endodontist performed the examinations over approximately two months to reduce recording errors. The exclusion criteria included cases without sufficient clinical or radiographical information, non-Saudi individuals under 18 years of age, and patients with bone metabolic disturbance, familial colon polyposis, and Gardner’s syndrome [2,4,5]. Moreover, the following radiopacities were excluded: characteristic mixed radiopaque-radiolucent areas of periapical cemental dysplasia, benign fibro-osseous lesions of periodontal ligament origin, widening of the lamina dura around the teeth due to malposition or trauma from occlusion, remaining roots of permanent or deciduous teeth, radiopaque tori or exostoses, and single radiopacities in edentulous regions, which may have been excessively ossified surgical sites.

**Figure 1:**
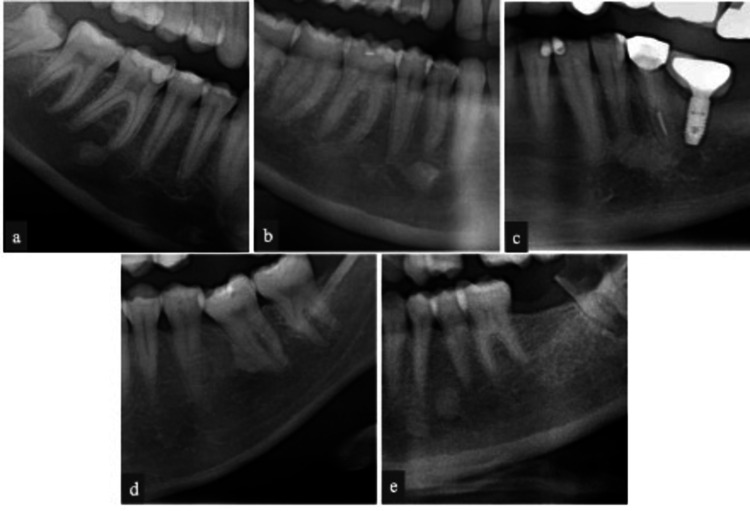
Panoramic radiographs showing osteosclerotic lesions related to teeth and root apices in different relations. (a) Apical, (b) separate, (c) apical interradicular, (d) interradicular, and (e) interradicular and separate.

Lesions were considered to be CO according to their radiographic appearance [10] and if chronic inflammation was present. Usually, these lesions surround the apices of teeth with deep carious lesions, root canal treatment, or large restorations. Lesions that were well defined without any inflammation that were not ruled out by the examples listed above were classified as IO [15]. The investigator recorded gender, age, tooth number, tooth location, tooth relation, shape, and border related to the lesions associated with CO and IO. The dental status used to differentiate the teeth involved with CO and IO was classified as deep caries, restoration, root canal treatment, edentulous, no lesion/disease found, or any related significant findings. The location of the lesion was categorized according to the jaw involved (i.e., maxilla or mandible) and the associated jaw region (i.e., anterior, premolar, and molar). The tooth relation was classified using the criteria by Geist and Katz [2]: (1) apical if the masses were dominantly around the root apices (Figure 1a); (2) separate if the radiopacities were apical to and clearly separated away from the teeth and lamina dura (Figure 1b); (3) apical and interradicular if the radiopacities were at the apices and exhibited significant extension between the roots (Figure 1c); (4) interradicular if the sclerotic tissue was restricted to the area between the roots (Figure 1d); and (5) interradicular and separate when it was limited to the area between the roots but separate from the lamina dura (Figure 1e). The shape of the lesion was categorized as round, oval, or irregular, while the lesion border was either well or ill-defined.

Statistical analysis

Statistical Package for Social Sciences Program version 25 (IBM Corp., Armonk, NY, USA) was used for data analysis. Descriptive analysis and chi-square test were used to analyze the lesion distribution according to gender, age, border, shape, localization, and dental relation. Qualitative data were presented as frequencies and percentages. Inferential data analysis was done using the chi-square test. Statistical significance was considered at P-values of <0.05.

## Results

This study evaluated a total of 1,000 panoramic radiographs, and 752 (495 females, 257 males) patients fulfilled the inclusion criteria. Age ranged between 18 and 90 years old, with a mean age of 40 years (±SD). Out of the 752 patients (100%), CO and IO radiopacities were present in 110 (14.6%) patients. CO was found in 44 (5.9%) patients, while IO was found in 66 (8.8%) patients. Out of the 44 cases of CO, 36 (81.8%) were females and eight (18.2%) were males. There was a statistically significant difference in the prevalence of CO between males and females (P = 0.02), as shown in Table 1. IO was found in 66 patients, of whom 45 (68.2%) were females and 21 (31.8%) were males, with no statistically significant difference regarding the prevalence of IO between males and females (P = 0.54) (Table 1).

**Table 1:** Frequencies (percentages) of idiopathic osteosclerosis and condensing osteitis according to gender. *: significant P-values.

	Total (n = 110)	Male (n = 29)	Female (n = 81)	P-value
Idiopathic osteosclerosis	66 (100%)	21 (31.8%)	45 (68.2%)	0.54
Condensing osteitis	44 (100%)	8 (18.2%)	36 (81.8%)	0.02*

Table 2 shows that both lesions were found more in patients who were in their second and third decades of life. However, there was no significant difference between different age groups and the prevalence of either CO or IO.

**Table 2:** Frequencies (percentages) of idiopathic osteosclerosis and condensing osteitis according to age groups.

Age (year)	Idiopathic osteosclerosis (n = 66)	P-value	Condensing osteitis (n = 44)	P-value
20–30	29 (43.9)	0.06	13 (29.5)	0.55
31–40	16 (24.2)		11 (25.0)	
41–50	13 (19.7)		9 (20.5)	
51–60	2 (3.0)		9 (20.5)	
61–70	4 (6.1)		1 (2.3)	
Above 70	2 (3)		1 (2.3)	

As shown in Table 3, all CO and IO lesions were located in the mandible. Both lesions were mostly found in the molar area (68.2%), followed by the premolar area (27.3%) and anterior area (4.5%). The difference in location was statistically significant (P = 0.000). Radiopacities with well-defined borders comprised 68.2%, while 31.8% had ill-defined borders. With regards to shape, 47.3% showed an irregular shape, 40.9% had a rounded shape, and 11.8% had an oval shape. Statistically significant differences were noted in the shape (P = 0.02) and the border (P = 0.001) of the lesions. Detailed distributions of CO and IO according to their location, shape, and border are presented in Table 3.

**Table 3:** Frequencies (percentages) of idiopathic osteosclerosis and condensing osteitis with respect to location, shape, and radiographic border. *: significant P-values.

Variable		Idiopathic osteosclerosis (n = 66)	Condensing osteitis (n = 44)	Total (n = 110)	P-value
Location (region)	Anterior	5 (7.6)	0	5 (4.5)	0.003*
	Premolar	24 (36.4)	6 (13.6)	30 (27.3)	
	Molar	37 (56.1)	38 (86.4)	75 (68.2)	
Shape	Oval	10 (15.2)	3 (6.8)	13 (11.8)	0.02*
	Round	32 (48.5)	13 (29.5)	45 (40.9)	
	Irregular	24 (36.4)	28 (63.6)	52 (47.3)	
Border	Well-defined	53 (80.3)	22 (50.0)	75 (68.2)	0.001*
	Ill-defined	13 (19.7)	22 (50.0)	35 (31.8)	

Regarding lesion-tooth relation, the majority of CO lesions (88.6 %) were present in the apical space of the associated teeth. The difference in the lesion-tooth relation was statistically significant (P = 0.000). In contrast, 59% of IO lesions were separate from the teeth, 29% were associated with root apices, and 12% were associated with the interradicular space (Table 4).

**Table 4:** Frequencies (percentages) of idiopathic osteosclerosis and condensing osteitis according to tooth relation.

	Idiopathic osteosclerosis (n = 66)	Condensing osteitis (n = 44)	P-value
Apical	19 (28.8%)	39 (88.6%)	<0.001
Apical and interradicular	1 (1.5%)	4 (9.1%)	
Interradicular	5 (7.6%)	0 (0.0%)	
Interradicular and separate	2 (3.0%)	0 (0.0%)	
Separate	39 (59.1%)	1 (2.3%)	

The majority of IO radiopaque lesions (80.3%) had no related significant dental findings, while CO was mainly associated with root canal-treated teeth in most cases (45.5%), followed by carious (36.4%) and restored (18.2%) teeth. The difference was statistically significant (P = 0.000). Table 5 illustrates some of the main dental findings associated with both lesions.

**Table 5:** Frequencies (percentages) of related dental findings associated with idiopathic osteosclerosis and condensing osteitis. *: significant P-values.

Variable	Idiopathic osteosclerosis (n = 66)	Condensing osteitis (n = 44)	P-value
Caries	3 (4.5)	16 (36.4)	0.000*
Restoration	1 (1.5)	8 (18.2)	
RCT	2 (3.0)	20 (45.5)	
Edentulous	7 (10.6)	0 (0.0%)	
No related findings	53 (80.3)	0 (0.0%)	

## Discussion

Our study found that the overall prevalence of IO and CO is 8.8% and 5.9%, respectively. The prevalence of IO and CO in the Saudi population falls within the range of other reported studies in the region, which ranged from 3% to 8%. Miloglu et al. reported the lowest prevalence of IO at 2.5% and CO at 0.81% [15]. On the other hand, Farhadi et al. showed the highest prevalence for IO (7.5 %) and CO (7.8%), which exceeded our findings in terms of CO [16]. None of the previous studies in the literature reported a higher rate of CO than IO [14-16], which is consistent with our findings. To our knowledge, this is the first study to examine the jawbones of a Saudi subpopulation and report the prevalence of CO and IO radiopacities.

This study focused only on adult participants with ages ranging from 18 to 90 years. Most participants who had positive findings were in their third decade of life. IO and CO lesions were observed with the highest frequency at ages ranging from 21 to 30 years, with a percentage of 42.4% and 27.3%, respectively. Our findings agree with Kawai et al. who reported that the third decade had the highest incidence of IO [13]. Therefore, the prevalence of IO and CO lesions in our subpopulation tended to be more in the younger population. On the contrary, Yeh et al. had a higher prevalence of CO lesions (26.7%) among the older ages (50-59 years) [17]. A possible interpretation could be related to high caries and pulpal infection incidence as age increases.

In our study, both IO and CO lesions were more prevalent among females. This finding corresponds with other studies that reported a higher prevalence of dense bone island (IO) among females than males [2,6,15]. McDonnell reviewed 107 patient records looking for radiopacities that resembled IO and concluded that 67% of the lesions occurred in females while only 33% occurred in males [6]. This is a similar finding in comparison to our study, where we found the prevalence of IO among females to be 68.2% and 31.8% for males. Moreover, CO had a higher rate of occurrence in females (81.8%) than males (18.2%) in our study. These results support the findings of Yeh et al. which suggested that the dominance of the female gender over the male gender in terms of lesion prevalence could be related to specific female hormones that affect bone growth [17]. For example, estrogen is an essential hormone needed for the maturation and regulation of bone turnover. Whenever there is any defect in the hormone regulation process, the result would be either bone resorption or bone deposition. Further investigation with a larger sample is needed to determine a strong correlation between gender and the occurrence of CO lesions.

The location of lesions concerning the jaws is a critical finding that describes the disease pattern in a certain population. Our findings show that both CO and IO lesions occurred only in the mandible. This is in agreement with the findings reported by Miloglu et al. who showed that 99% of IO and 100% of CO lesions occurred in the lower jaw [15]. This occurrence of both lesions in the mandible can be attributed to the fact that there are some anatomical differences regarding the bone structure, blood supply, and anatomy of the jaws. Another explanation can be related to the vague appearances of lesions in the maxilla that the examiner can mistakenly overlook in the radiographs. On panoramic radiographs, the superimposition of anatomic components in the maxilla presents more challenges than the mandible, which can obstruct the visibility of any conditions resembling CO or dense bone island [7].

IO tends to be separated from the surrounding tissue. We observed that the most common relation of the lesion with the surroundings was being separated, as shown in 59.1% of the cases. However, this is not the case for CO, as we found that most lesions were associated with the apical part of the tooth (88.6%). These results are consistent with the data reported by Miloglu et al. who found that IO mostly had a separate relation to dental tissues (40%), and CO was associated with the apical part of the tooth (58%) [15]. This can be explained by the fact that IO is a developmental condition of normal bone, and its relation to a stimulant is rare. On the other hand, CO is believed to occur more in the apical position due to irritants originating from the diseased tooth or other possible etiological factors such as traumatic occlusion. Both conditions appeared to occur more in the posterior area, specifically the mandible molar area. McDonnell found that IO was mostly present in the first molar area (49%) [6]. These findings contradict Giest et al. who found the premolar area had the most IO lesions (17.9%) [2]. Caries, traumatic occlusion, and pulpal infections, which occur more commonly in posterior teeth than in anterior teeth, can be linked to the higher prevalence of CO lesions in the molar region [10].

Radiographically, the shape of IO appeared round in 48.5% of the lesions, which is consistent with the findings of Miloglu et al. who found a round shape in 68% of the cases [15]. However, our results contradict their findings in terms of the shape of CO, as we found the most common shape among the Saudi population to be of an irregular pattern (63.6%) not rounded.

CO was associated more with root canal-treated teeth (45%), whereas most of the IO lesions had no significant dental findings. This emphasizes the previously mentioned finding that the condition of IO is developmental rather than a reactionary condition. Moreover, it supports the idea that CO is usually associated with either an inflamed or a necrotic pulp, which can greatly aid the dental clinician in diagnosing pulpally involved teeth and reaching an accurate endodontic diagnosis.

Based on our findings, we believe that IO is caused by developmental changes in normal bone architecture that are not related to local stimuli. The lesions may appear at any age or location, with no gender preference. Other than diagnosis and identification, IO normally does not require any treatment. However, the lesions must be followed up regularly to confirm an accurate clinical diagnosis. Endodontic therapy or extraction of the associated tooth may be required if the associated root resorbs over time, which is uncommon. CO lesions, on the other hand, are defined as a bone reaction to infection, which is commonly caused by a carious tooth, a deeply restored tooth, or a tooth with an inflamed pulp.

Finally, the analysis of the conditions undertaken here has extended our knowledge into the different radio-opaque lesions that can be discovered routinely in the clinics. Moreover, it is one of the limited studies that compare IO and CO lesions simultaneously. However, knowledge of the characteristics and prevalence of IO and CO concluded from our study may be considered as limited as it is based on a retrospective cross-sectional study collected from one area using convenient sampling, which limits the generalization of our findings. Future longitudinal studies done in various locations may report specific evidence regarding the origin and development of the lesions in Saudi Arabia.

## Conclusions

This is the first study to report the prevalence of IO and CO in a Saudi subpopulation from Jeddah, Saudi Arabia. The prevalence of IO and CO was 8.8% and 5.9%, respectively. These findings demonstrate a low prevalence of these lesions but show that they are more frequent among females in their third decade and primarily found in the mandibular molar region.
